# Bioactive Potential of Chitosan–Oleic Acid Nanoparticles Loaded with Lemon Peel Essential Oil for Topical Treatment of Vulvovaginal Candidiasis

**DOI:** 10.3390/molecules29235766

**Published:** 2024-12-06

**Authors:** Faten M. Ibrahim, Eman Samy Shalaby, Mohamed F. Abdelhameed, Radwa H. El-Akad, Kawkab A. Ahmed, Mohamed S. Abdel-Aziz, El Sayed El Habbasha, Cristina V. Rodrigues, Manuela Pintado

**Affiliations:** 1 Medicinal and Aromatic Plants Research Department, Pharmaceutical and Drug Industries Research Institute, National Research Centre, Cairo P.O. Box 12622, Egypt; 2 Pharmaceutical Technology Department, Pharmaceutical and Drug Industries Research Institute, National Research Centre, Cairo P.O. Box 12622, Egypt; dremanshalaby_nrc@hotmail.com; 3 Pharmacology Department, Medical Research and Clinical Studies Institute, National Research Centre, Dokki, Giza P.O. Box 12622, Egypt; fayed.nrc@gmail.com; 4 Pharmacognosy Department, Pharmaceutical and Drug Industries Research Institute, National Research Centre, Dokki, Giza P.O. Box 12622, Egypt; radwa.hassan.elakad@gmail.com; 5 Department of Pathology, Faculty of Veterinary Medicine, Cairo University, Giza P.O. Box 12211, Egypt; kawkababdelaziz@yahoo.com; 6 Microbial Chemistry Department, Biotechnology Research Institute, National Research Centre, Cairo P.O. Box 12622, Egypt; mohabomerna@yahoo.ca; 7 Field Crops Research Department, National Research Centre, Cairo P.O. Box 12622, Egypt; sayedhabasha@yahoo.com; 8 CBQF—Centro de Biotecnologia e Química Fina, Laboratório Associado, Escola Superior de Biotecnologia, Universidade Católica Portuguesa, Rua Diogo Botelho 1327, 4169-005 Porto, Portugal; civrodrigues@ucp.pt

**Keywords:** citrus byproducts, lemon essential oil, antimicrobial therapies, bioactive compounds, chitosan–oleic acid nanoparticles, vulvovaginal candidiasis, molecular docking

## Abstract

The rising incidence of vulvovaginal candidiasis (VVC) has been leading to the development of alternative antifungal therapies. This study aimed to develop a topical chitosan–oleic acid nanoparticle (CH-OA-NP) cream loaded with lemon peel essential oil (LPEO) for VVC treatment. The characterization of the optimal nanoparticle formulation (F4: 10 g/L CH, 2:1 OA/LPEO ratio) showed high encapsulation efficiency, stability, and controlled release. Moreover, it was characterized regarding its particle size, polydispersity index, zeta potential, and chemical/morphological profile. LPEO-related compounds (e.g., eriodictyol) were identified through LC-ESI-QqTOF-HRMS in the cream matrix, suggesting the preservation of LPEO potential bioactivities after formulation. In silico docking of 12 LPEO metabolites revealed that compounds such as citronellic acid exerted inhibitory effects against several inflammation-associated enzymes (e.g., 14-α-Demethylase). In vitro antimicrobial tests demonstrated remarkable activity against *Candida albicans*, Gram-negative (e.g., *Escherichia coli*), and Gram-positive (e.g., *Staphylococcus aureus*) bacteria. *In vivo* studies in a rat model of VVC revealed significant antifungal, anti-inflammatory, and immunomodulatory effects of the LPEO-CH-OA-NP cream (5% and 10%), leading to reduced MDA, MPO, and IL-1β levels and increased GSH activity. This novel formulation potentially offers a promising alternative therapy for VVC, addressing the current antifungal therapies’ limitations, counteracting drug resistance.

## 1. Introduction

Vulvovaginal candidiasis (VVC) is a prevalent gynecological condition characterized by the inflammation of the vaginal mucosa, primarily caused by *Candida albicans.* While VVC can affect women of all ages, it is most common during the reproductive years, with approximately 75% of women experiencing at least one episode in their lifetime [1]. In addition, the prevalence of VVC has been increasing in developing countries [2]. *Candida* species are commensal yeasts that commonly colonies the skin and mucosal surfaces of humans, particularly the gastrointestinal and reproductive tracts [3]. However, overgrowth of these yeasts, particularly *C. albicans*, can disrupt the normal vaginal microbiota, leading to the development of VVC. The two major classes of antifungal drugs used to treat fungal infections, including VVC, are azoles (e.g., clotrimazole, fluconazole, itraconazole, and ketoconazole) and polyenes (e.g., amphotericin B and nystatin) [4]. Azoles exert a fungistatic effect, inhibiting fungal growth by preventing ergosterol synthesis, a major component of the fungal membrane. However, prolonged or high-dose azole therapies can lead to host toxicity and the emergence of drug resistance [5]. The increasing prevalence of drug-resistant *Candida* species, coupled with the potential side effects associated with prolonged azole treatments, leads to challenges regarding the efficient treatment of VVC, among other fungal infections [3]. Therefore, there is growing interest in exploring complementary and alternative treatment options for VVC.

Essential oils are interesting compounds known for their diverse bioactivities, including antibacterial, antifungal, antiviral, antioxidant, and potential anticancer and immune-modulatory effects. Moreover, they have also been traditionally used for their analgesic, anti-inflammatory, spasmolytic, and local anesthetic properties [6]. Lemon peel essential oil (LPEO), derived from *Citrus limon*, in particular, has demonstrated promising antimicrobial activity. This oil is mainly composed of limonene, β-pinene, γ-terpinene, and citral (neral and geranial), which contribute to its therapeutic potential [7]. Furthermore, it was reported that LPEO might exert its antifungal effects by disrupting the permeability and integrity of the fungi cell membrane. This disruption leads to the leakage of intracellular components, compromising cellular viability [8].

To enhance the efficacy and delivery of LPEO for VVC treatment, studies have been exploring sustained-release systems using biodegradable polymers like chitosan, meaning that these systems promote a controlled release of the drug over time, while preventing its degradation [9]. Chitosan is a cationic polysaccharide composed of glucosamine and N-acetylglucosamine units, being produced by the partial deacetylation of chitin [9]. In addition, this compound was reported to be biocompatible, biodegradable, and to hold antimicrobial properties. Moreover, its mucoadhesive nature, attributed to the presence of hydroxyl and amino groups, makes it particularly suitable for topical applications, including vaginal drug delivery [9]. In this way, chitosan interacts with the mucosal surface through electrostatic interactions, facilitating drug permeation via the paracellular route [9].

Chitosan-based nanocarriers have emerged as promising vehicles for overcoming the limitations of hydrophobic bioactive compounds (e.g., poor water solubility and low bioavailability). This is attributed to chitosan biocompatibility and biodegradability and the presence of reactive amino groups along its polymer chains. These amino groups facilitate versatile interactions with various amphiphilic and/or anionic biopolymers and molecules, making chitosan an exceptional material for encapsulation and enhancing the aqueous solubility of hydrophobic compounds [10]. Several materials, such as tamarind [11], chondroitin sulphate [12], gelatin [13], zein [14], and cellulose [15], have been combined with chitosan for the encapsulation of bioactive compounds. Oleic acid, for instance, is an omega-9 monounsaturated fatty acid present in high concentrations in vegetable oils and nuts (e.g., olive oil and peanuts) [10]. This compound holds both anionic carboxylic groups and hydrophobic alkyl chains, which allows easy interaction with cationic chitosan, while providing a hydrophobic region, which is useful for the entrapment of lemon peel essential oil within the nanoparticles. Since this technique is driven by the self-assembly of molecules, compounds such as emulsifiers, surfactants, or crosslinking agents are not required. Moreover, this reaction can be performed at room temperature, making it advantageous for industrial applications [10,12].

This work aimed to develop a novel topical cream for VVC treatment, by encapsulating LPEO into chitosan–oleic acid nanoparticles (CH-OA-NPs). This self-assembling nanoparticle system was designed to enhance LPEO delivery to infected tissues, while enhancing tissue adhesion, thereby improving its potential bioactivities (e.g., antifungal, antioxidant, and anti-inflammatory), targeting candidiasis symptoms. Moreover, this study carried out a comprehensive characterization approach, utilizing techniques such as high-resolution liquid chromatography/mass spectroscopy and in silico docking studies to uncover the properties and mechanisms of action of the developed formulation.

While essential oils, including LPEO, have been explored for their antimicrobial properties [7,8], their therapeutic application remains unexplored due to limitations like poor solubility and stability, particularly in hydrophobic environments. Moreover, the integration of LPEO into a chitosan–oleic acid nanoparticle system to address these challenges that is specifically designed for the treatment of VVC has not been reported. This work contributes to the field by developing a novel topical cream formulation that combines the bioadhesive and antimicrobial properties of chitosan with the sustained-release and stability-enhancing benefits of oleic acid nanoparticles. Furthermore, the formulation was thoroughly evaluated, providing insights into its therapeutic potential. To our knowledge, this is the first study to explore the encapsulation of LPEO in CH-OA-NPs and its targeted application for VVC, offering a promising alternative to conventional antifungal therapies, which have been limited by issues such as resistance and toxicity. Overall, the findings reported in this study highlight the potential of the LPEO-loaded CHI-OA-NPs cream as an effective alternative for the topical treatment for VVC.

## 2. Results and Discussion

### 2.1. Characterization of the Developed LPEO-Loaded CH-OA-NPs

#### 2.1.1. LPEO Encapsulation Efficiency

For all the tested formulations (F1–F6), LPEO was successfully encapsulated in the CH-OA complex nanoparticles. The LPEO encapsulation efficiency into the CH-OA-NPs ranged between 56.16 to 86.71% (Table 1, LPEO content (%)). In addition to the percentage uptake, the total amount of LPEO encapsulated was determined based on the phenolic content calculation, where 1 g of phenolic content (gallic acid equivalents) corresponds to 5 mg of LPEO. For the most efficient formulation (F4), which exhibited an encapsulation efficiency (EE) of 86.71 ± 0.97%, this corresponds to a total encapsulation of approximately 493.7 mg of LPEO. On the other hand, the least efficient formulation (F2) encapsulated 477.4 mg of LPEO, corresponding to an EE of 56.16 ± 0.18%. Therefore, a higher concentration of CH significantly (*p* < 0.05) led to a higher encapsulation efficiency. This can be justified by the assembling of a thicker chitosan layer around the LPEO molecules [16]. Moreover, the increase in the LPEO concentration showed a noticeable increase in the encapsulation efficiency. Similar results were reported by Gooneh-Farahani et al. [17].

#### 2.1.2. Particle Size, Polydispersity Index (PDI), and Zeta Potential Assessments

All formulations of the nanoparticles’ (F1–F6) particle size, PDI, and zeta potential were characterized (Table 1).

Regarding the particle size, the values were determined using the dynamic light scattering (DLS) technique, which measures the hydrodynamic diameter of the nanoparticles in aqueous dispersion, giving results between 234.14 ± 0.11 and 757.70 ± 4.93 nm. It was observed that increasing the CH concentration led to an increase in the particle size, which corroborates previous literature reports [18]. The small particle size obtained with a lower concentration of CH was partly due to the decreased viscosity of the CH solution, which holds improved solubility for a more efficient gelation process. Moreover, at higher concentrations of CH, the particles tend to aggregate, forming larger particles [19]. The nanoparticles’ zeta potential presented positive values for all formulations, ranging between 53.2 ± 0.8 and 99.5 ± 1.5 mV, indicating stable nanosystems. The nanoparticles tend to remain stably dispersed when charged due to the electric repulsion between them [20]. Moreover, since CH carried a high positive charge, its adsorption increased the positive density of the systems [21]. Therefore, the increase in the CH concentration can increase zeta potential values, which has also been reported in other studies [21]. The PDI values ranged between 0.248 ± 0.060 and 0.773 ± 0.113. In this way, only F1, F2, F4, and F5 presented a PDI value lower than 0.5, meaning a narrow and uniform particle size distribution [22].

To proceed with the next assessments, the optimum formulation was chosen. In this way, since F4 shows the highest encapsulation efficiency (86.71 ± 0.97%), reasonable particle size (442.54 ± 0.91 nm), and zeta potential (68.6 ± 4.4 mV) and the lowest PDI (0.248 ± 0.06), it was selected for further assessments and incorporation into the cream.

#### 2.1.3. Fourier Transform Infrared Spectroscopy (FT-IR) Analysis

**Figure 1:**
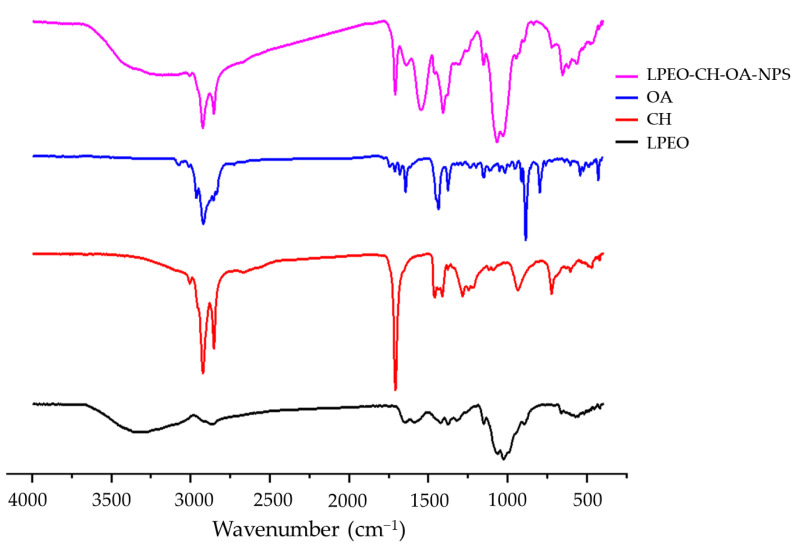
FTIR spectra of oleic acid (OA), chitosan (CH), lemon peel essential oil (LPEO), and the optimal nanoparticle formulation (LPEO-CH-OA-NPs).

The FTIR spectra of LPEO, CH, OA, and F4 (LPEO-CH-OA-NPs) were assessed to characterize their chemical structure and interactions between components (Figure 1). The main peaks reported for LPEO appear at 2965, 1435, and 1147 cm^−1^. The peak at 2965 cm^−1^ corresponds to CH_2_ symmetric and symmetric vibrations, while the peak at 1435 cm^−1^ corresponds to C-H symmetric and a symmetric bends [23]. Moreover, the characteristic peaks of individual components (LPEO, CH, and OA) are similar to the FTIR profiles reported in the literature, indicating their high purity degree and suitability nanoparticles formulations. The formation of the LPEO-CH-OA-NPs is confirmed, by comparing the nanoparticles and the crude LPEO spectra. FTIR spectra showed changes in the peak intensity and the position of LPEO after inclusion in the nanoparticles. The LPEO peaks at 2965 cm^−1^ and 1435 cm^−1^ are shifted or fused in the IR spectra of the LPEO-CH-OA-NPs, which indicates interactions between CH, OA, and LPEO and the successful inclusion of LPEO into the nanoparticles. On the other hand, despite being diffused, the LPEO-CH-OA-NP spectrum showed the characteristic peaks of both CH and OA. In this way, the intensity of the chitosan-related peak at 2955 cm^−1^ decreased upon the nanoparticles’ formulation. Moreover, it is important to note that LPEO peaks in the nanoparticle formulation did not significantly shift or vanish, and no new peaks were observed, potentially indicating that the extract is still intact and capable of exerting its therapeutic effect after inclusion in the nanoparticles.

#### 2.1.4. Transmission Electron Microscopy (TEM) Analysis

**Figure 2:**
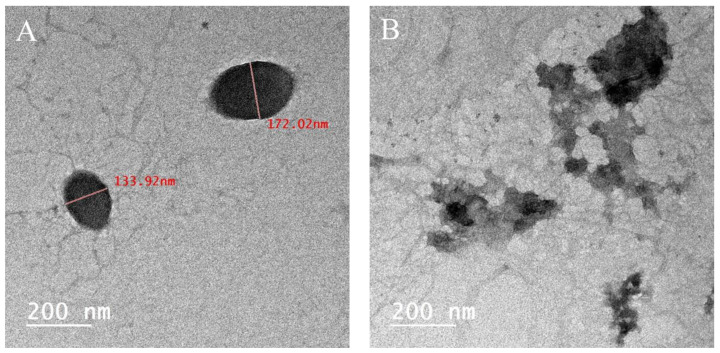
TEM micrograph of the selected optimal LPEO-CH-OA-NPs showing their size and shape (**A**) and their aggregation behavior (**B**).

A TEM analysis was conducted to examine the morphology of the optimal LPEO-CH-OA-NPs. The micrographs showed that the particles were spherical with a droplet size below 200 nm, confirming their nanoscale (Figure 2A), as well as their aggregation behavior (Figure 2B). Moreover, the significant difference observed between the sizes measured by DLS (Table 1) and TEM can be explained by the drying process involved in TEM sample preparation. Nanoparticles often undergo shrinkage during drying due to the evaporation of water and other volatiles, leading to smaller particle dimensions, which emphasizes the complementary nature of these techniques, and the importance of considering both sets of measurements to fully characterize the nanoparticles. While DLS provides data regarding the particles’ behavior in their native hydrated state, TEM assesses their physical dimensions post-drying [24,25].

#### 2.1.5. In Vitro Antimicrobial Activity Assessment of LPEO and LPEO-CH-OA-NPs

Vulvovaginal candidiasis is an infection caused by *Candida* species [26]. Moreover, among the infection, several opportunistic microorganisms might proliferate, leading to other health issues. Moreover, among women with acute vulvovaginal candidiasis, *Candida albicans* accounts for 80–90% of the isolated fungal species [27]. Hence, the antimicrobial activity of LPEO and LPEO-CH-OA-NPs at 5, 20, and 50 mg/mL was assessed against *S. aureus*, *E. coli*, and *C. albicans*, through the determination of the minimum inhibitory concentration (MIC) and minimum microbicidal concentration (MMC) (Table 2).

It was concluded that the tested LPEO and LPEO-CH-OA-NPs were capable of inhibiting the growth of the tested Gram-positive and Gram-negative bacteria as well as the yeast strain. The results revealed that the LPEO showed low MIC values against all tested microbes, namely 312.50 ± 2.57, 156.25 ± 3.12, and 39.06 ± 0.79 µg/mL for *S. aureus*, *E. coli*, and *C. albicans*, respectively. Regarding MMC results LPEO showed values of 625.00 ± 5.23, 312.50 ± 4.81, and 78.13 ± 1.09 µg/mL for *S. aureus*, *E. coli*, and *C. albicans*, respectively. The results for LPEO-CH-OA-NPs demonstrated a dose-dependent antimicrobial activity against all tested microorganisms, as shown by the MIC and MMC values. Therefore, higher concentrations of LPEO-CH-OA-NPs resulted in lower MIC and MMC values, with the 50 mg/mL concentration showing the most effective results across all microorganisms. The MIC values for this concentration were 9.77 ± 1.05, 9.78 ± 0.94, and 9.78 ± 0.92 µg/mL for *S. aureus*, *E. coli*, and *C. albicans*, respectively, while the MMC values were 19.53 ± 1.42, 19.53 ± 1.65, and 9.78 ± 0.92 µg/mL for the same microorganisms. Moreover, in Table 2, it is observed that the MIC-to-MMC ratio varied among the tested organisms. These differences might be because fungal cell walls (e.g., in *C. albicans*) are structurally different from bacterial cell walls, potentially requiring a smaller concentration increase for total eradication compared to bacterial species, like *S. aureus* and *E. coli*. Additionally, the results demonstrated that the LPEO-CH-OA-NP formulation (F4) exhibits significantly stronger antimicrobial activity compared to LPEO alone, as evidenced by the lower MIC and MMC values across all tested microorganisms. This improvement can be attributed to the unique properties of nanoparticles, which enhance the delivery and efficacy of LPEO through multiple mechanisms. Firstly, the nanoscale size of the LPEO-CH-OA-NPs allows for a higher surface-area-to-volume ratio, enabling more efficient interaction with microbial cells. Moreover, it is important to consider the potential contribution of chitosan’s intrinsic antimicrobial properties to the observed efficacy of LPEO-CH-OA-NPs. The polycationic nature of chitosan in the nanoparticles might enhance binding to the negatively charged surfaces of bacterial and fungal cells, leading to the greater disruption of microbial membranes compared to LPEO alone. Also, the encapsulation of LPEO in CH-OA-NPs provides the controlled and sustained release of LPEO, maintaining an effective concentration of the bioactive compounds over time. Furthermore, by protecting LPEO from degradation, encapsulation ensures that its antimicrobial compounds remain active and might exert their effects more effectively. In addition, it has been reported in the literature that the possible mechanisms wherein LPEO interfere with bacterial and fungal proliferation may involve (i) the disintegration of the bacterial outer membrane or phospholipid bilayer, (ii) the alteration of the fatty acid composition, (iii) the increase in membrane fluidity resulting in the leakage of potassium ions and protons, (iv) interference with glucose uptake, and (v) the inhibition of enzyme activity or cell lysis [28,29,30].

### 2.2. Characterization of the Formulated LPEO-CH-OA-NP Topical Cream

#### 2.2.1. Rheology Assessment

**Figure 3:**
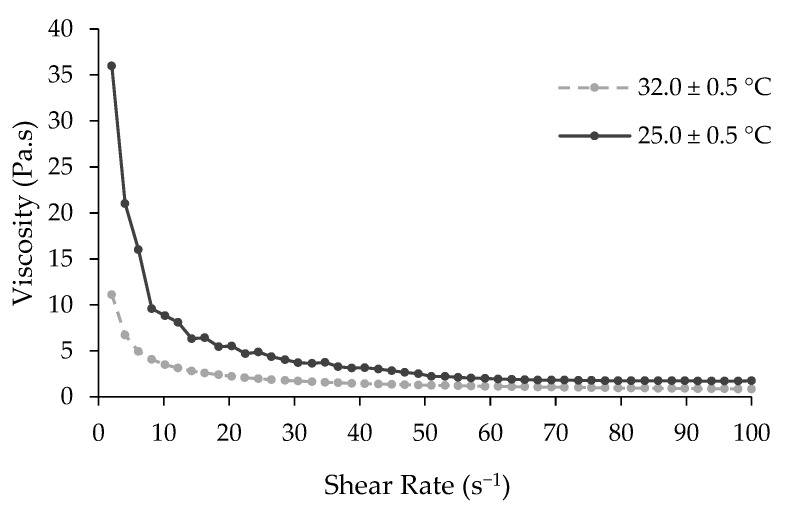
Rheogram of the developed LPEO-CH-OA-NP topical cream. The plot is presented as an average of duplicates.

For ideal application, the topical cream formulation based on LPEO-CH-OA-NPs must be easily spreadable and nondripping in nature. Therefore, after its formulation, the rheology of the cream was assessed. Figure 3 shows the viscosity of the cream as a function of the shear rate, at 25.0 ± 0.5 °C and 32.0 ± 0.5 °C. It can be concluded that the viscosity of the cream decreased with higher temperatures.

Higher temperatures reduced the solid fraction, enhancing flow and deformability, and leading to lower viscosity [31]. Moreover, the results revealed that the cream exhibited a Newtonian shear thinning flow behavior, meaning a decrease in viscosity with an increase in the shear rate. When the formulation is subjected to a shear force, its network structure breaks down leading to a gradual viscosity decrease. This property is beneficial for this kind of formulation, since it allows for the cream’s removal from the container and facilitates spreading and application on the skin.

#### 2.2.2. In Vitro Release Profile

**Figure 4:**
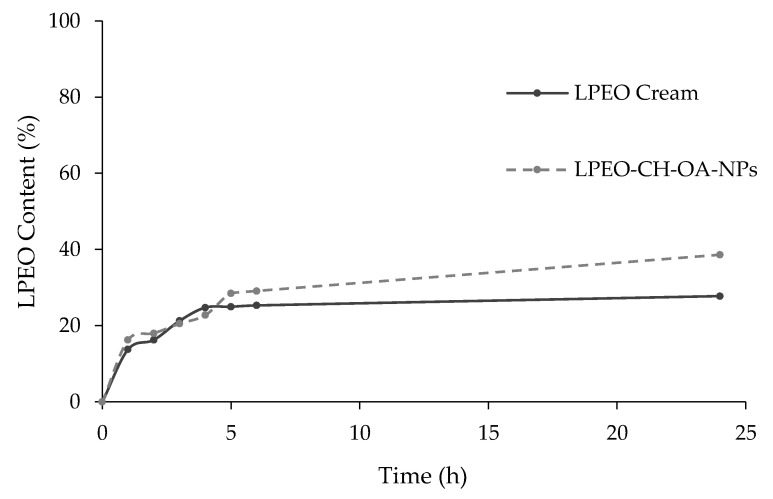
In vitro release profiles of LPEO from the LPEO-CH-OA-NPs and from the cream loaded with LPEO-CH-OA-NPs (LPEO cream).

The release profiles of LPEO from the LPEO-CH-OA-NP and LPEO creams are shown in Figure 4. Both samples showed slow and controlled release after 24 h. The sustained release of active substances can be achieved by tuning the diffusional path by cross-linking and/or forming complexes with the chitosan amino and hydroxyl groups, as described for a variety of molecules [19]. On its aggregate in solution, the CH–OA can provide more protection to the formed system and slow drug release [32]. Moreover, chitosan provides higher encapsulation and a diffusional barrier that further enhances the controlled release behavior of LPEO from nanoparticles and the cream [33]. The inclusion of the LPEO-CH-OA-NPs in the cream formulation led to a noticeably slower release of LPEO, which is in accordance with similar reports made [34,35,36].

#### 2.2.3. LPEO-Derived Compounds: Tentative Identification by LC-ESI-QqTOF-HRMS

After performing an extraction to the developed cream, LC-ESI-QqTOF-HRMS was employed to tentatively identify LPEO-derived compounds. The mass spectrometric analysis resulted in the identification of 12 compounds, namely terpenes, organic acids, and flavonoids (Table 3). Also, the minimal error observed in the mass measurement (mDa) suggests high accuracy in the identification of the compounds. The identified compounds include well-known constituents of LEPO, which are recognized for their bioactive properties, such as antimicrobial, antioxidant, and anti-inflammatory properties [37]. The presence of bioactive terpenes, such as terpinolene, limonene, and α-terpinene, alongside flavonoids like eriodictyol, suggests that LPEO holds significant potential for therapeutic applications regarding VVC, even after formulation [37].

#### 2.2.4. In Silico Antifungal Activity of the Identified LPEO-Derived Compounds

In order to further understand the mechanisms by which LPEO exerts its antifungal activity, an in silico study was conducted to correlate the observed activity with the identified metabolites via LC-ESI-QqTOF-HRMS analysis.

Azole and morpholine antifungal drugs target several enzymes that are critical for the growth and metabolism of *C. albicans*. For this study, four enzymes were selected: (i) 14α-demethylase (PDB ID: 5TZ1) required for ergosterol biosynthesis; (ii) ∆-14-sterol reductase (PDB ID: 4QUV), essential for the stability of the cell membrane; (iii) 1,3-β-glucansynthase (PDB ID: 1EQC), which catalyzes the synthesis of (1,3)-β-glucan, a major polymer component of the cell wall; and (vi) thymidylate synthase (PDB ID: 5UIV), which catalyzes the intracellular de novo formation of thymidylate thus essential for DNA biosynthesis. The docking model validation was performed through the redocking of co-crystalized ligands into their respective enzymes. The obtained values of root mean square deviation (RMSD, ≤1 °A) (Table 4) indicate reliable modelling. Miconazole, a known antifungal drug, was used as a reference to compare docking scores [38,39].

**Figure 5:**
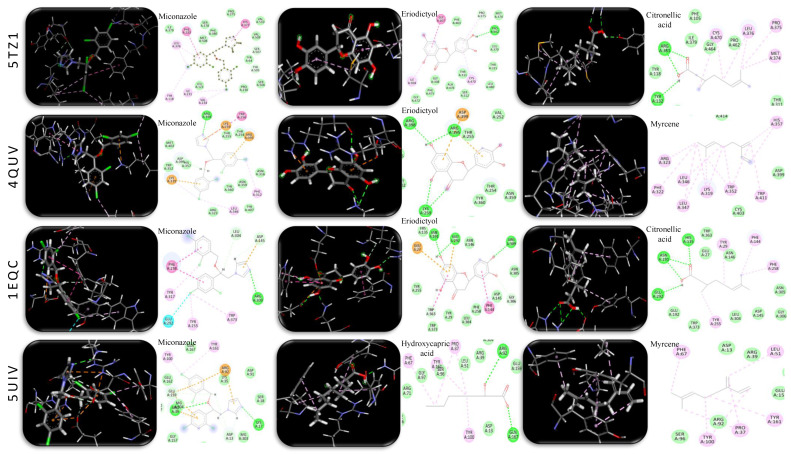
3D and 2D molecular docking simulations of the miconazole control drug and selected metabolites detected in LPEO-CH-OA topical cream extract that showed high binding affinity evidenced by their docking scores through hydrophilic (green) and hydrophobic (purple) interactions with 14-α-demethylase (PDB ID: 5TZ1), ∆-14-sterol reductase (PDB ID: 4QUV), 1,3-β-glucansynthase (PDB ID: 1EQC), and thymidylate synthase (PDB ID: 5UIV) resembling their co-crystalized ligand interaction with active sites.

The nature of the metabolites docked herein varied between polar acids/flavonoids and nonpolar monoterpenes; thus, diverse hydrophilic and hydrophobic bonding with active sites was observed, matching co-crystalized ligand interactions (Table 4 and Figure 5). Overall, the highest docking scores were reported for eriodictyol, citronellic acid, and hydroxycapric acid, which showed improved inhibitory effects against all tested enzymes, when compared to miconazole. This was achieved through hydrophilic interactions via carboxyl and hydroxyl moieties as well as Pi-Pi and Pi-Alkyl hydrophobic interactions (Table 4). Moreover, when compared to miconazole (−6.121 kcal mol^−1^), monoterpenes, particularly myrcene, limonene, and terpinene, exhibited a higher inhibitory activity against thymidylate synthase (PDB ID: 5UIV) through Pi-Alkyl hydrophobic interaction, as evidenced by docking scores at −6.18, −5.52, and −5.609 kcal mol^−1^, respectively. The fact that other monoterpenes as pinene (−3.97 kcal mol^−1^) and camphene, (−3.94 kcal mol^−1^) showed the weakest binding affinity to the same receptor indicated that a minimum of two pi-bonds are necessary for a positive interaction (Table 4). On the other hand, eriodictyol exhibited its highest docking score (−7.077 kcal mol^−1^) against 1,3-β-glucansynthase (PDB ID: 1EQC) exceeding miconazole (−6.979 kcal mol^−1^). It was indeed observed that co-crystalized ligand hydrophilic interaction with GLU A: 27, GLU A: 292, ASN A: 191, GLU A: 27, and HIS A: 135 is crucial for enzyme inhibition, which is herein reported for eriodictyol, citronellic acid, hydroxycapric acid, and linalool oxide (Table 4 and Figure 5).

The results obtained in this study match the published literature on the antifungal activity of LPEO and its components [29,40,41]. However, to the best of our knowledge, this is the first work reporting the possible different mechanisms by which its components exert their activity.

### 2.3. In Vivo Properties Assessment of the LPEO and LPEO-CH-OA-NPs Topical Cream

#### 2.3.1. Acute Toxicity Evaluation: Hematological and Biochemical Analysis

##### Effect on Leukocytes and Differential Leukocytic Count

**Figure 6:**
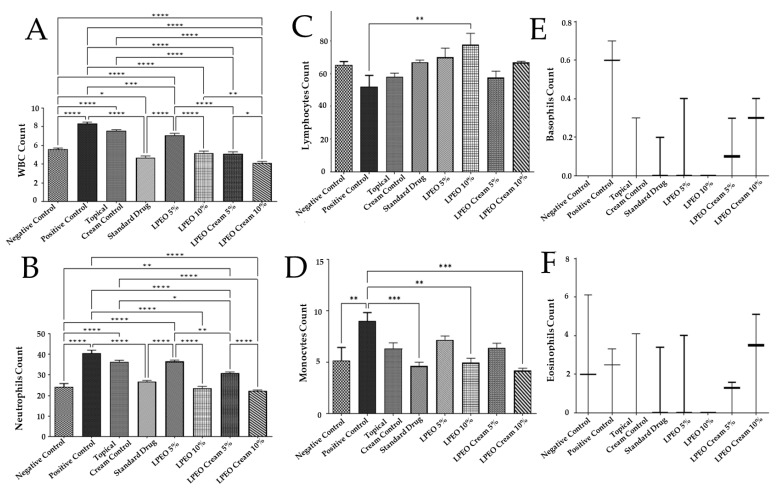
Effect of treatment with LPEO (5 and 10%) and LPEO cream (5 and 10%) on (**A**) WBCs, (**B**) neutrophils, (**C**) lymphocytes, (**D**) monocytes, (**E**) basophils, and (**F**) eosinophil counts in vulvovaginal candidiasis in rats. Data are expressed as mean ± SD, *n* = 5. Statistical analysis was carried out by one-way analysis of variance (ANOVA) followed by Tukey’s multiple comparison test. * *p*  ≤  0.05, ** *p*  ≤  0.01, *** *p*  ≤  0.001, **** *p*  ≤  0.0001.

When immunity is compromised or the vaginal microbiome is disrupted, recurrent mucosal *Candida* infections may occur, potentially leading to candidiasis [42]. Previous reports have shown elevated white blood cell (WBC) counts in vaginal infections [43]. In this study, the intravaginal inoculation of *Candida albicans* induced a significant increase in the WBC count (*p*  ≤  0.0001) compared to the negative control group, indicating the presence of infection, inflammation, injury, or even, immune system disorders. However, treatment with LPEO nanocream at concentrations of 5% and 10% significantly reduced the WBC counts compared to the same concentrations of free LPEO and the positive control group (*p*  ≤  0.0001). The reduction in WBC count followed the following trend: LPEO cream 10% < LPEO cream 5% < LPEO 10% < LPEO 5%. Notably, no significant changes in the WBC count were observed in the topical cream control group compared to the positive control group (Figure 6A), suggesting that the LPEO nanocream formulation was more effective in reducing inflammatory responses associated with *Candida albicans* infections.

Polymorphonuclear neutrophils are short-lived effector cells, essential for phagocytosing pathogens and mediating tissue damage, thereby serving as the first line of defense against microbial invasions [43]. Neutrophils play a critical role against bacterial and fungal infections by migrating to sites of tissue injury, where they form dense groups. Their robust pathogen resistance capability can, however, result in significant collateral damage, exacerbating immune responses, tissue loss, and potentially leading to organ dysfunction [43]. During this study, the differential cell count followed a similar pattern to the WBC counts. The intravaginal inoculation of *Candida albicans* caused a significant (*p* ≤ 0.0001) increase in neutrophil count compared to the negative control group, highlighting the critical role of neutrophils in host defense against invasive candidiasis [44]. Treatment with both LPEO and LPEO nanocream at 5% and 10% significantly reduced neutrophil counts (*p* ≤ 0.0001) compared to the positive control group. Additionally, increasing the LPEO dose from 5% to 10% in both the free LPEO and nanocream formulations further decreased the neutrophil counts. The reduction in neutrophil levels followed the following order: LPEO cream 10% < LPEO 10% < LPEO cream 5% < LPEO 5%. Notably, the application of topical cream control did not result in any significant changes in neutrophil count when compared to the positive control group (*p* > 0.05) (Figure 6B). This suggests that the LPEO formulations, particularly in nanocream form, are effective in modulating neutrophil responses during *Candida albicans* infection.

Intravaginal inoculation with *Candida albicans* resulted in a significant decrease in lymphocyte count (*p* ≤  0.0001) compared to the negative control group, indicating the critical role of lymphocytes during the early stages of infection (first 5 days), likely due to lymphocytic adhesion to *Candida* [45]. Treatment with both free LPEO and LPEO cream at 5% and 10% doses significantly increased lymphocyte counts (*p* ≤  0.01) compared to the positive control group (Figure 6C).

Monocytes, another key leukocyte involved in the inflammatory process, migrate from the bloodstream into tissues and mature into macrophages over 2–3 days. Previous studies have shown that *C. albicans* triggers the recruitment of a large number of macrophages to the vaginal tissue [30]. In this study, the intravaginal inoculation of *Candida* significantly increased monocyte counts (*p*  ≤  0.05) compared to the negative control group. However, treatment with LPEO nanocream at the 10% dose significantly reduced monocyte counts (*p*  ≤  0.01) compared to the positive control group, highlighting the efficacy of higher doses of the nanocream in mitigating *Candida*-induced inflammation (Figure 6D).

There were no significant changes in basophil or eosinophil counts across normal, infected, and treated groups (Figure 6E,F), suggesting that these leukocytes were not majorly involved in the inflammatory response or treatment effects in this model of *Candida* infection.

Overall, these results emphasize the potential of high-dose LPEO cream in modulating immune responses, particularly by restoring lymphocyte and monocyte counts, thereby alleviating inflammation and infection caused by *Candida albicans*.

##### Effect on Platelets

**Figure 7:**
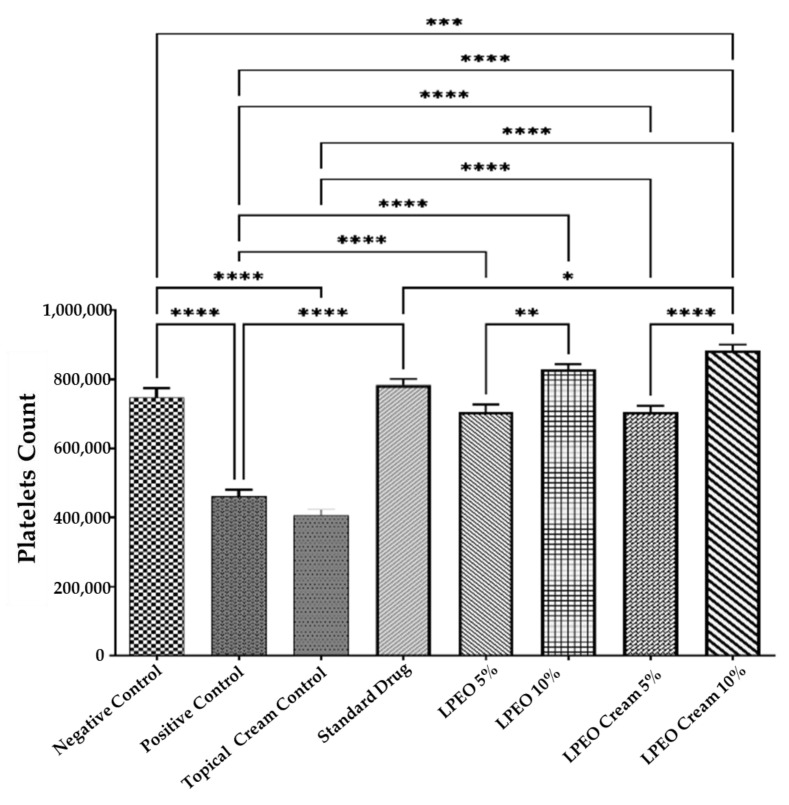
Effect of treatment with LPEO (5 and 10%) and LPEO cream (5 and 10%) on the platelet count in VVC in rats. Data are expressed as mean ± SD, *n* = 5. Statistical analysis was carried out by one-way analysis of variance (ANOVA) followed by Tukey’s multiple comparison test. * *p*  ≤  0.05, ** *p*  ≤  0.01, *** *p*  ≤  0.001, **** *p*  ≤  0.0001.

The intravaginal inoculation with *Candida albicans* resulted in a significant reduction (*p*  ≤  0.0001) in platelet count compared to the normal control group. However, treatment with both LPEO and the nanocream at 5% and 10% concentrations significantly increased platelet counts (*p*  ≤  0.0001) relative to the positive control group. The increase in platelet count followed the following order: LPEO cream 10% > LPEO cream 5% > LPEO 10% > LPEO 5%. No significant changes in platelet count were observed in the group treated with the topical cream control, indicating that the formulation alone had no effect (Figure 7). These findings highlight the efficacy of LPEO, particularly in its nanocream form, in restoring platelet levels following *Candida infection*.

##### Hematological Parameters Assessment

**Figure 8:**
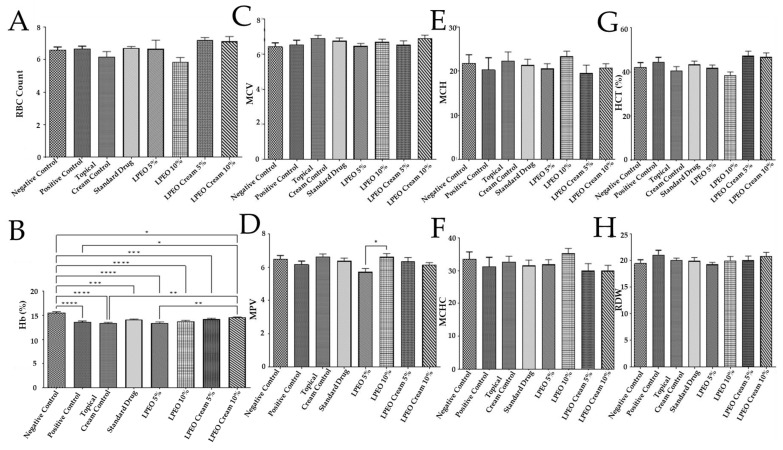
Effect of treatment with LPEO (5 and 10%) and LPEO cream (5 and 10%) on (**A**) RBCs, (**B**) hemoglobin, (**C**) MCV, (**D**) MPV, (**E**) MCH, (**F**) MCHC, (**G**) HCT, and (**H**) RDW in VVC in rats. Data are expressed as mean ± SD, *n* = 5. Statistical analysis was carried out by one-way analysis of variance (ANOVA) followed by Tukey’s multiple comparison test. * *p*  ≤  0.05, ** *p*  ≤  0.01, *** *p*  ≤  0.001, **** *p*  ≤  0.0001.

The hematological parameter (red blood cells count (RBCs), hemoglobin percentage (Hb%), mean corpuscular volume (MCV), mean platelet volume (MPV), mean corpuscular hemoglobin (MCH), mean corpuscular hemoglobin concentration (MCHC), hematocrit percentage of red blood cells in the blood (HCT%), and red cell distribution width (RDW)) are references for inflammation and are widely used in clinics [30]. The intravaginal *Candida* inoculum did not exert any significant changes when compared to the normal control group in all hematological parameters except hemoglobin (Hb). In this way, the positive control significantly (*p*  ≤  0.0001) decreased Hb when compared to the negative control group. Moreover, the application of the topical cream control, LPEO, or the LPEO cream did not result in any significant changes in the hematological parameters, except for the group treated with the LPEO cream 10%. In this group, a significant increase in hemoglobin levels (Hb%) was observed (*p* ≤  0.05) compared to the positive control group (Figure 8A–H). This suggests that the LPEO nanocream 10% formulation may have a unique ability to enhance hemoglobin levels, while other treatments had no notable impact on hematological outcomes.

##### Oxidative Stress Assessment

**Figure 9:**
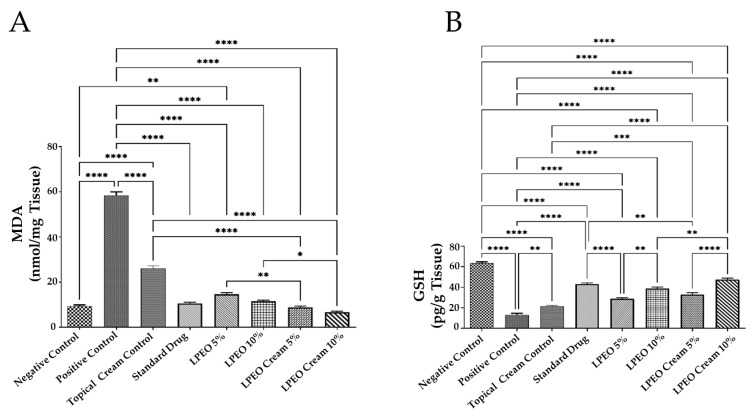
Effect of treatment with LPEO (5 and 10%) and LPEO cream (5 and 10%) on the vulvovaginal wash content of (**A**) MDA and (**B**) GSH in VVC in rats. Data are expressed as mean ± SD, *n* = 5. Statistical analysis was carried out by one-way analysis of variance (ANOVA) followed by Tukey’s multiple comparison test. * *p*  ≤  0.05, ** *p*  ≤  0.01, *** *p*  ≤  0.001, **** *p*  ≤  0.0001.

Oxidative stress plays a significant role in various physiological conditions and numerous diseases, including VVC [46]. The intravaginal inoculation of *Candida albicans* led to a significant increase in malondialdehyde (MDA) levels (*p* ≤ 0.0001) and a drastic decrease in glutathione (GSH) levels (*p* ≤ 0.0001) compared to the negative control group. In contrast, the administration of LPEO and LPEO cream at both concentrations (5 and 10%) significantly reduced MDA levels (*p* ≤ 0.0001) and restored GSH levels (*p* ≤ 0.0001) in a dose-dependent manner compared to the positive control group (Figure 9A,B). Notably, the LPEO cream at 10% significantly restored MDA and GSH levels in vaginal tissue infected with *Candida*, returning them to a normal state (*p* < 0.05). The slight antioxidant activity observed in the topical cream control formulation may be attributed to the chitosan content in the nanoparticles, which also possess antioxidant properties [47]. These findings align with those of Campelo et al., who demonstrated the antioxidant effects of LPEO [48].

##### Effect on Inflammatory Markers

**Figure 10:**
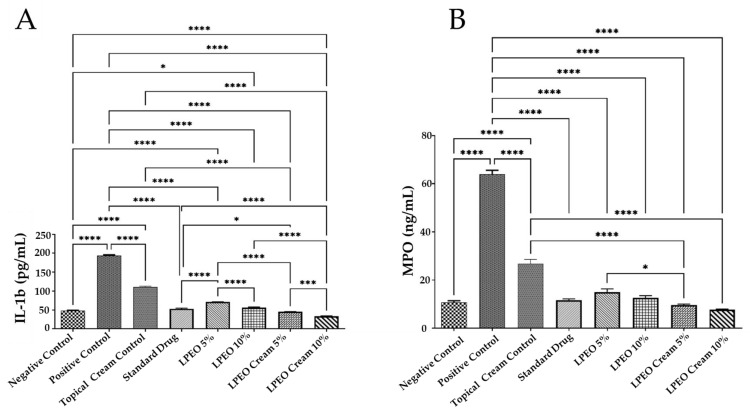
Effect of treatment with LPEO (5 and 10%) and LPEO cream (5 and 10%) on the vulvovaginal wash content of (**A**) MPO and (**B**) IL-1b in VVC in rats. Data are expressed as mean ± SD, *n* = 5. Statistical analysis was carried out by one-way analysis of variance (ANOVA) and followed by Tukey’s multiple comparison test. * *p*  ≤  0.05, *** *p*  ≤  0.001, **** *p*  ≤  0.0001.

VVC spread is mainly mediated by the hosts’ immune system. Overgrowth of *Candida albicans*, along with its yeast-to-hyphae transition and the production of candidalysin, triggers epithelial cells to release antimicrobial peptides and proinflammatory cytokines and chemokines, such as interleukin-1 beta (IL-1β). This, in turn, leads to the recruitment of non-protective neutrophils, creating a hyperinflammatory environment that is primarily responsible for VVC symptoms [49]. In this study, genital mucosal fluid from rats was analyzed for distinct immune mediators, including the proinflammatory cytokine IL-1β and a neutrophil marker myeloperoxidase (MPO). The intravaginal inoculation of *Candida albicans* resulted in a significant increase in both MPO and IL-1β levels (*p* ≤ 0.0001) compared to the negative control group. However, treatment with LPEO and LPEO nanocream, at 5% and 10%, significantly reduced MPO and IL-1β levels (*p* ≤ 0.0001) compared to the positive control group (Figure 10A,B). The LPEO nanocream was particularly effective in restoring MPO and IL-1β levels to normal physiological states. The LPEO nanocream demonstrated a more pronounced reduction in MPO levels in infected tissues compared to free LPEO in a dose-dependent manner. In this way, the encapsulation of LPEO into the nanosystem was able to significantly enhance its anti-inflammatory properties. Moreover, the slight anti-inflammatory activity shown by the topical cream control formulation could be attributed to its chitosan content, as it also holds anti-inflammatory properties [47]. Previous studies demonstrated that the significant anti-inflammatory effects showed by citrus essential oils are probably due to the presence of limonene in their composition [50].

#### 2.3.2. Antimicrobial Evaluation

**Figure 11:**
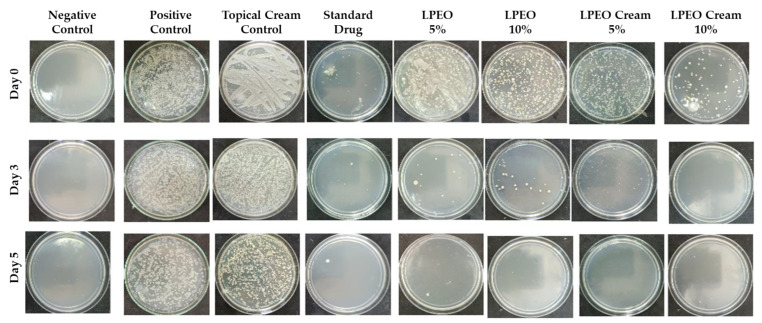
Microbial growth of vaginal fluids samples recorded at day 0, 3, and 5 within different groups.

**Figure 12:**
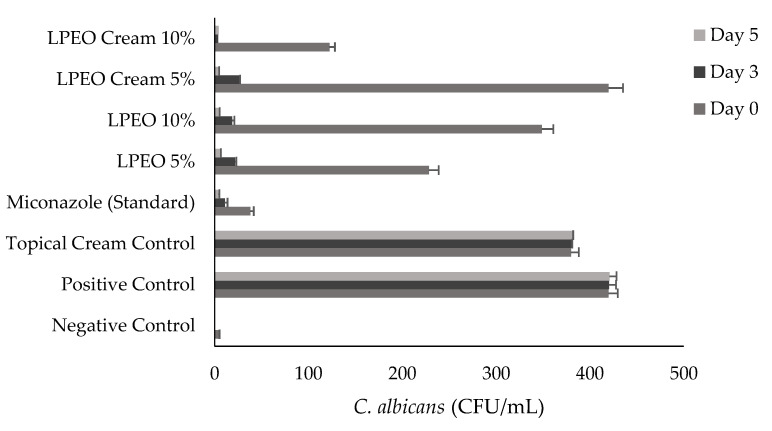
Number of colony-forming units of *Candida albicans* (CFU/mL) in control, treated, and untreated rats’ groups. Data are presented as mean ± SD. One-way ANOVA was used for data analysis (*n* = 3, *p* ≤ 0.05).

The data presented in Figure 11 and Figure 12 illustrate the colony-forming units (CFU) of *Candida albicans* across all groups after days 0, 3, and 5 of treatment with LPEO (5 and 10%) or LPEO cream (5 and 10%). In the healthy control group (Group I, negative control), no microbial growth was detected from the swabs. In contrast, Group II, which represented untreated candidiasis (positive control), showed over 400 *Candida* colonies. Group III, treated with a topical cream control, exhibited no significant reduction in *C. albicans* growth compared to the positive control. Group IV, treated with the reference drug miconazole, demonstrated a significant reduction in infection within the first 24 h, with further decreases observed throughout the 5-day treatment period, leading to fewer than 10 colonies by the end of the assessment. Groups V and VI, treated with free LPEO at 5% and 10%, respectively, showed only a slight reduction in microbial colonies after the first 24 h. However, both groups exhibited a significant reduction in microbial growth by the end of the 5-day treatment, leading to the complete inhibition of *C. albicans* growth, comparable to the negative control. Similarly, Groups VII and VIII, which were treated with LPEO nanocream at 5% and 10%, respectively, showed a marked decrease in microbial growth, achieving near-complete inhibition after 96 h. In particular, Group VIII (LPEO cream 10%) demonstrated a more pronounced reduction in microbial growth within the first 24 h compared to Group VII (LPEO cream 5%).

#### 2.3.3. Histopathological Assessments

**Figure 13:**
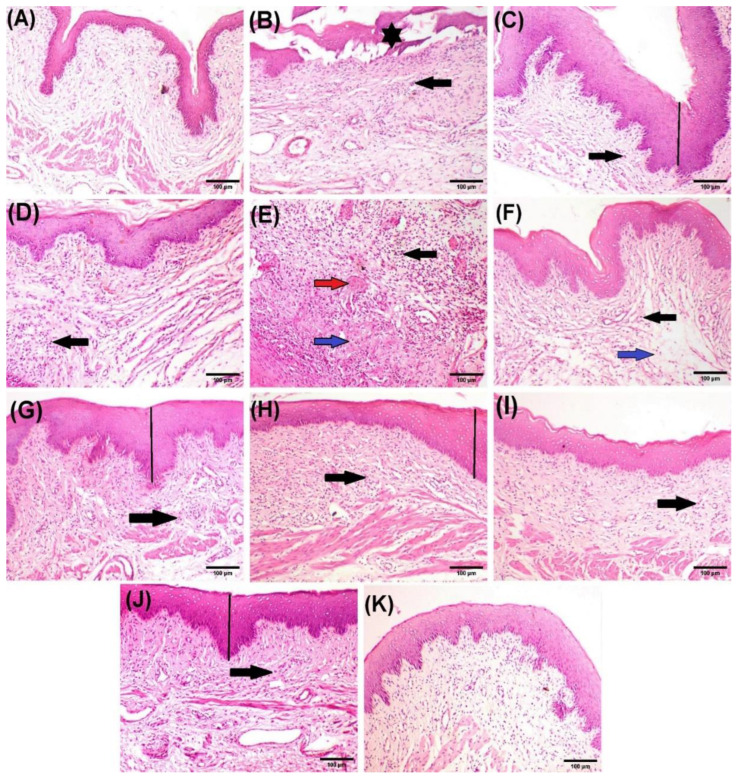
Photomicrographs of rat vaginal tissue after hematoxylin and eosin staining. (**A**) Negative control group showing normal histoarchitecture of vaginal tissue. (**B**–**E**) Positive control group, showing necrosis and detachment of mucosa (star), hyperplastic mucosal epithelium (line), heavy submucosal inflammatory cells infiltration (black arrow), submucosal edema (blue arrow), and congested blood vessels (red arrow). (**F**) Topical cream control group, showing moderate submucosal inflammatory cells infiltration (black arrow) associated with edema (blue arrow). (**G**) Reference drug showing focal hyperplastic mucosa (line) and mild inflammatory cells infiltration (black arrow). (**H**) LPEO 5% group showing focal hyperplastic mucosa (line) and moderate inflammatory cells infiltration (black arrow). (**I**) LPEO 10% group showing mild inflammatory cell infiltration (black arrow). (**J**) LPEO cream 5% group showing slight hyperplasic mucosa (line) and mild submucosal inflammatory cell infiltration (black arrow). (**K**) LPEO cream 10% group showing normal histological features of the vaginal tissue. (Scale bar, 100 µm).

The microscopic examination of vaginal tissue from the negative control group showed a healthy stratified squamous epithelium and no signs of inflammation. The submucosa contained dense connective tissue fibers and well-organized blood vessels (Figure 13A). In contrast, vaginal tissue from the infected positive control group exhibited marked histopathological damage, including necrosis and the detachment of the mucosal layer (Figure 13B), the hyperplastic mucosal epithelium (Figure 13D), significant submucosal inflammatory cell infiltration (Figure 13B–E), submucosal oedema, and congested blood vessels (Figure 13E). The vaginal tissue of rats treated with the topical cream control showed moderate submucosal inflammatory cell infiltration along with oedema (Figure 13F). In comparison, rats treated with the reference drug (miconazole) displayed focal hyperplasia of the mucosa and mild inflammatory cell infiltration (Figure 13G). Similarly, sections from rats treated with LPEO 5% showed focal hyperplasia of the mucosa and moderate infiltration of inflammatory cells (Figure 13H). Treatment with LPEO 10% resulted in only mild inflammatory cell infiltration (Figure 13I). Moreover, the application of LPEO cream led to significant improvement in vaginal lesions. Rats treated with LPEO cream 5% showed only slight mucosal hyperplasia and mild submucosal inflammatory cell infiltration (Figure 13J), while those treated with LPEO cream 10% exhibited normal histological features, with no signs of inflammation or tissue damage (Figure 13K). The histopathological scores for the different experimental groups are summarized in Table 5.

## 3. Materials and Methods

### 3.1. Materials

Fresh lemon peels (*Citrus limon*) were sourced from a local market in Egypt to extract their essential oil. Additionally, chia oil was obtained following the method by El Makawy et al. [51]. Chitosan (deacetylation degree > 90%, viscosity ranging from 30 to 100 cps, and an average molecular weight of 250,000 g/mol) was acquired from Glentham Life Sciences (Corsham-Wiltshire, UK). The oleic acid was sourced from Sigma-Aldrich (St. Louis, MO, USA). Polawax™ NF was obtained from Croda International (East Yorkshire, UK). Propylene glycol was purchased from Spectrum Chemicals (New Brunswick, NJ, USA). Potassium mono-hydrogen phosphate and di-potassium hydrogen phosphate were obtained from El Nasr Pharmaceutical Chemicals (Cairo, Egypt). MPO, IL-1β, MDA, and GSH activity levels were evaluated using enzyme-linked immunosorbent assay (ELISA) kits from Thermo Fisher Scientific Inc. (Waltham, MA, USA). All chemicals used were used as received, and they were of analytical grade.

### 3.2. Extraction of Lemon Peel Essential Oil (LPEO)

The lemon peel essential oil was extracted through hydro-distillation using a Clevenger-type apparatus for 3 h, as described in the Egyptian pharmacopoeia [52].

### 3.3. Development of LPEO-Loaded Chitosan–Oleic Acid Nanoparticles (CH-OA-NPs)

The preparation of nanoparticles based on chitosan (CH) oleic acid (OA) complexes was carried out following the method described by Kuroiwa et al. [53] with slight modifications. CH powder was combined with 100 mL of dH_2_O and dissolved in 20 mL of 2% (*v*/*v*) acetic acid while stirring. The solution was adjusted to a final volume of 200 mL, resulting in CH concentrations of 5 and 10 g/L. The CH solutions were then filtered through filter paper. OA and LPEO were mixed in 2:1, 1:1, and 1:2 ratios, using 99.5% (*v*/*v*) ethanol. Subsequently, 2 mL of the OA-LPEO solution was added gradually to 20 mL of CH solution, while stirring (400 rpm, room temperature (RT)). In total, six formulations (F1–F6) were achieved and tested for the nanoparticles (Table 6). The solutions were stored at RT until further use.

### 3.4. Characterization of the Developed LPEO-CH-OA-NPs

#### 3.4.1. LPEO Encapsulation Efficiency

The LPEO content of the developed LPEO-CH-OA-NPs was determined spectrophotometrically through the Folin–Ciocalteu method. Therefore, the nanoparticles were thoroughly dissolved in dimethyl sulfoxide (DMSO), followed by stirring for 24 h, and filtration [53,54]. Afterwards, 5 mL of a 10% (*v*/*v*) Folin–Ciocalteu reagent was mixed with 1 mL of the LPEO-CH-OA-NPs extract. Then, 4 mL of 7.5% (*w*/*v*) sodium carbonate was added to the solution, followed by a 24 h incubation, in the dark, at RT. Then, the solution was homogenized using a vortex and its absorbance was assessed at 745 nm, using a Spectrophotometer (UV-2401 PC, Shimadzu Co., Kyoto, Japan) [55]. Gallic acid was used as a standard for the calibration curve. The drug content was calculated by equating 1 g of phenolic content to 5 mg of LPEO.

#### 3.4.2. Particle Size, Polydispersity Index (PDI), and Zeta Potential Assessments

The measurement of particle size and the analysis of zeta potential for the developed nanoparticles were performed at 25 ± 2 °C, with a 1:100 dilution in dH_2_O [56] through photon correlation spectroscopy and dynamic light scattering (DLS), with the Malvern Zetasizer ZS (Malvern Instruments, Ltd., Worcestershire, UK).

#### 3.4.3. Fourier Transform Infrared Spectroscopy (FT-IR) Analysis

The chemical integrity and possible chemical interaction between LPEO and CHI-OA nanoparticles components were analyzed using a FT-IR spectrophotometer (JASCO 6100, Tokyo, Japan). Each solid-state sample, namely chitosan and the freeze-dried LPEO-CH-OA-NPs, was mixed with KBr and compressed under a pressure of 200 kg/cm^2^ for 2 min using a hydraulic press to form compact disks. Also, the liquid samples (LPEO, OA) were directly analyzed. All samples were scanned against a blank KBr pellet background between 4000 and 400 cm^−1^. Each spectrum was acquired by an average of 32 scans in transmission mode [57,58].

#### 3.4.4. Transmission Electron Microscopy (TEM) Analysis

The morphological characteristics of selected nanoparticle systems were assessed through TEM (JEM-2100, JEOL Co., Akishima, Japan) to evaluate structural attributes including lamellarity, shape, and size.

### 3.5. In Vitro Antimicrobial Activity Assessment of LPEO and LPEO-CH-OA-NPs

The minimum inhibitory concentration (MIC) and minimum bactericidal concentration (MBC) of LPEO (50 mg/mL) and LPEO-CH-OA-NPs (5, 20, 50 mg/mL) were assessed against *Staphylococcus aureus* ATCC 6538 (Gram-positive), *Escherichia coli* ATCC 25933 (Gram-negative), and *Candida albicans* ATCC 10231 (yeast). The microbial strains were inoculated in Mueller–Hinton medium (BBL, Darmstadt, Germany), followed by incubation at 35 °C for 24 h. The cells were then collected by centrifugation (4000 rpm, 15 min), under sterile conditions, and re-suspended in phosphate-buffered saline (PBS) to obtain stock cultures. Serial dilutions were performed under aseptic conditions to reach an optical density between 0.5 and 1.0 (500 nm). The number of colony-forming units (CFU) was then assessed, to ensure a concentration of 5.0 × 10⁶ CFU/mL. The resazurin solution was prepared by dissolving 67.5 mg of the reagent in 10 mL of sterile dH_2_O [56].

To a 96-well plate, 100 µL of each test material was added to the first plate row; the, 100 µL of Mueller–Hinton broth was added to the remaining wells. Serial dilutions were performed. Subsequently, 100 µL of bacterial suspension (5 × 10^6^ CFU/mL) was added to each well [55]. Duplicates for each concentration of each tested solution were made. Negative control (only medium) and positive control (medium and each strain) of microbial growth were made. The plates were then incubated at 37 °C for 18–24 h.

After, 10 µL of resazurin solution was added to each testing and control well, and an additional 4 h of incubation was carried out. The color change was then evaluated visually. Any shifts in color from purple to pink or colorless were noted. The lowest concentration at which a color change was observed was recorded as the MIC value [57]. In addition, the MBC was defined as the concentration of each sample that displayed no bacterial growth on freshly inoculated agar plates [59]. Each test condition was performed in duplicate.

### 3.6. Formulation and Characterization of the LPEO-CH-OA-NPs Topical Cream

#### 3.6.1. Formulation of the LPEO-CH-OA-NPs Topical Cream

The vaginal cream matrix was prepared as described in previous studies [59,60], using final concentrations of 10% (*w*/*v*) polawax, 2% (*w*/*v*) (M.P. 50 °C) chia oil, and 5% (*w*/*v*) propylene glycol. The oil phase (polawax and chia oil) was homogenized. Propylene glycol and water were mixed and heated (10 °C above the wax melting point). The aqueous phase was added to the oil phase and stirred continuously until it cooled down. During the cool-down phase, the LPEO-CH-OA-NPs were added after the temperature reached 40 °C. The pH value of the formulation was adjusted to 4.5 to mimic the vaginal physiological pH value.

#### 3.6.2. Rheology Assessment of the LPEO-CH-OA-NP Topical Cream

The rheological properties of the prepared LPEO-CH-OA-NP topical cream were determined using a parallel-plate rheometer (Physica MCR 301, Anton Paar, Ostfildern, Germany), to determine shear viscosity as a function of shear rate. A total of 50 measurements were carried out with shear rates between 1 s^−1^ and 250 s^−1^, at 25 ± 0.5 and 32 ± 0.5 °C [56]. The plot was acquired from the average of duplicates.

#### 3.6.3. In Vitro Release Profile of the LPEO-CH-OA-NPs

The in vitro release profiles of LPEO from nanoparticles and LPEO-CH-OA loaded cream were assessed by the dialysis [61]. An amount equivalent to 15 mg of oil for both the LPEO-CH-OA-NPs and the cream was added into presoaked dialysis membranes (Dialysis tubing cellulose membrane, molecular weight cut-off 12,000–14,000, Sigma-Aldrich Co., St. Louis, MO, USA). The dialysis was carried out against 100 mL of acetate buffer (pH 5.5) with 10% ethanol (*v*/*v*) solution [61,62], in a shaking water bath (Memmert, SV 1422, Schwabach, Germany) at 32 ± 0.5 °C, 100 rpm. The samples were withdrawn every 2 h, up until the 24 h time point, to assess the LPEO concentration spectrophotometrically. The release profile of LPEO from selected NPs and cream formulations was compared to free LPEO solution containing an equivalent quantity of the oil. The cumulative percentage of LPEO released was determined as the ratio between the quantity of total polyphenols released (Cf) and the initial quantity of total phenolic compounds inserted in the dialysis membrane (Ci) (Equation (1)) [63]. All measurements were conducted in triplicate.

LPEO Release Quantity (%) = Cf/Ci × 100(1)

#### 3.6.4. LPEO-Derived Compounds Analysis by LC-ESI-QqTOF-HRMS

##### Compound Extraction from the Topical Cream Formulation

To analyze each cream composition, they were submitted to an extraction method based on the one described by Mapoung et al. [64], with some modifications. Briefly, the extraction process was carried out using a 50% (*v*/*v*) ethanol solution. Therefore, 0.5 g of each sample was placed in different 50 mL Falcon tubes lined with aluminum foil. Subsequently, 10 mL of the ethanol solution was added to each tube, followed by vigorous mixing for 3 min at room temperature (25 °C). Afterwards, the samples were sonicated using an ultrasonic homogenizer (Ultrasonic Processor Sonics VCX 130, Seoul, Republic of Korea) at 37% intensity for 10 min, with intervals for cooling on ice every 5 min. Then, each cream sample underwent centrifugation (4 °C, 5000 rpm, for 20 min). The resulting supernatant was filtered using filter paper and stored at −80 °C for further analysis.

##### Compounds Tentative Identification by LC-ESI-QqTOF-HRMS

The previously obtained extracts were analyzed by LC-ESI-UHR-QqTOF-MS, according to the method reported by Vilas-Boas et al. [65]. Briefly, the separation was performed in a UHPLC UltiMate 3000 Dionex (Thermo Scientific, Waltham, MA, USA), coupled to an ultrahigh-resolution, Qq-time-of-flight (UHR-QqTOF) mass spectrometer with 50,000 full-sensitivity resolution (FSR) (Impact II; Bruker Daltonics, Bremen, Germany). The separation was accomplished with an Acclaim RSLC 120 C18 column (100 mm × 2.1 mm, 2.2 μm) (Dionex, Sunnyvale, CA, USA). The injection volume was 5 μL. The mobile phases consisted of (A) 0.1% aqueous formic acid and (B) acetonitrile with 0.1% of formic acid, and the gradient elution conditions were 0 min, 0% B; 10 min, 21.0% B; 14 min, 27% B; 18.30 min, 58%; 20.0 min, 100%; 24.0 min, 100%; 24.10 min, 0%; and 26.0 min, 0% at a flow rate of 0.25 mL/min. Parameters for MS analysis were set using negative ionization mode with spectra acquired over a range from *m*/*z* 20 to 1000. The parameters were as follows: capillary voltage, 3.0 kV; drying gas temperature, 200 °C; drying gas flow, 8.0 L/min; nebulizing gas pressure, 2 bar; collision RF, 300 Vpp; transfer time, 120 μs; and prepulse storage, 4 μs. Post-acquisition internal mass calibration used HCOONa clusters, delivered by a syringe pump at the start of each chromatographic analysis. High-resolution mass spectrometry was used to identify the compounds. The elemental composition for the compound was confirmed according to accurate mass and isotope rate calculations designated mSigma (Bruker Daltonics, Billerica, MA, USA). The accurate mass measurement was within 5 mDa of the assigned elemental composition, and mSigma values of <20 provided confirmation. Compounds were identified based on their accurate mass [M−H]^+^. One independent analysis was performed in each triplicate extract obtained for each methodology.

#### 3.6.5. In Silico Antifungal Activity of LPEO-Derived Compounds

The study was performed on AutoDock (AD) inbuild vina PyRx 0.8 version software and Discovery Studio 2021. The X-ray crystal structure of the target proteins: 14-α-Demethylase (PDB ID: 5TZ1), ∆-14-Sterol reductase (PDB ID: 4QUV), 1,3-β-glucansynthase (PDB ID: 1EQC), and thymidylate synthase (PDB ID: 5UIV) were obtained from Protein Data Bank (PDB) (https://www.rcsb.org/, accessed on 14 February 2024). The ligands’ 3D structures were downloaded from PubChem database [66].

Protein preparation included the removal of water particles, the omission of extra chains, protonation, and energy minimization using MMFF94 force field [38,67]. Energy-minimized ligands (.sdf) included a control reference drug (miconazole) and 12 metabolites identified in LPEO via HR-LC/MS analysis of vaginal cream. The redocking of co-crystalized ligands was performed to validate the docking protocol.

The visualization and analysis of binding interactions were performed using Discovery Studio 2021. Binding energy score (−kcal/mol) and root mean square deviation (RMSD) values were considered for evaluating the docking results.

### 3.7. In Vivo Assays on Mice and Rats

#### 3.7.1. Acute Toxicity Assay on Mice

This assay was carried out according to the method described by El Fadaly et al. [68] Two-month-old male and female Swiss albino mice weighing an average of 20.4 g were used for this assay. The animals were housed in groups of five and allowed a five-day acclimation period before the start of any experiments. All procedures were conducted under OECD guideline no. 402 [69].

Before the assessment, the dorsal trunk fur of the mice was removed to create a hair-free surface of at least 10% of the body surface area. This facilitated the application of the selected LPEO-CH-OA-NP cream formulations or the free LPEO, which were applied topically at a dosage of 2000 mg/kg. The formulations were held in place using a porous gauze dressing and non-irritating tape for a 24 h exposure period. Following application, animals were monitored closely for 14 days for any changes in fur, eyes, behavior, and signs of toxicity. The LPEO-CH-OA-NP cream formulations and the free LPEO were concluded to be safe up to a dose of 2000 mg/kg, and appropriate dosages for further experimentation were determined based on these results.

#### 3.7.2. Vaginal Infection Model and Experimental Design on Rats

##### Animal Model

A rat model was chosen for this assay due to its accuracy in research on pathogen identification and host defense regarding fungal infections [43]. All procedures were carried out under OECD guideline no. 402 for the ethical treatment of animals [69].

##### Surgical Procedure and Infection Induction

Rats underwent bilateral ovariectomy surgery to induce a pseudoestrus state. The procedure was performed under anesthesia by intraperitoneal injection of pentobarbital sodium (3.0 mL/kg). Firstly, a longitudinal abdominal incision was made to expose the ovaries, located between the lower edge of the free ribs and the iliac crest. Next, the ovarian artery and vein were ligated before ovary removal. Finally, the muscle layers were sutured using resorbable sutures, and the skin was closed with nylon 4-0 sutures (non-absorbable, natural, multifilament, black suture/SK94019F4, Ethilon, Ethicon, Inc., Raritan, NJ, USA).

Post-operatively, the animals received antibiotics to prevent infection. The pseudoestrus state was maintained via subcutaneous injections of estradiol benzoate (Misr Co. for Pharm. Ind., Cairo, Egypt). Six days post-estradiol administration, rats were inoculated intravaginally with 0.1 mL of a *Candida albicans* suspension (10^9^ CFU/mL in 50 μM phosphate buffer, pH 7). Infection confirmation occurred 24 h post-inoculation by culturing serially diluted vaginal fluid samples on Sabouraud dextrose agar containing 50 mg/mL chloramphenicol. Rats exhibiting ≥ 10^3^ CFU/mL were considered infected.

##### Experimental Groups

Animals were randomly divided into eight groups (*n* = 10/group). Rats in Group I (negative control) received daily saline applications of 5 mL/kg and underwent a sham procedure. Group II (positive control) was inoculated with *Candida albicans*, as previously described. Group III was constituted of bilaterally ovariectomized rats, which received daily applications of cream without the testing bioactives (LPEO or NPs) [70]. Group IV contained bilaterally ovariectomized rats with daily applications of miconazole 2% (*v*/*v*), a commercial fungicide drug (standard). Group V was constituted of bilaterally ovariectomized rats, with daily applications of 5% (*v*/*v*) free LPEO. Group VI held bilaterally ovariectomized rats with daily applications of 10% (*v*/*v*) free LPEO. Group VII contained bilaterally ovariectomized rats, with daily applications of 5% (*v*/*v*) LPEO-CH-OA-NP cream. Group VIII contained bilaterally ovariectomized rats with 10% (*v*/*v*) LPEO-CH-OA-NP cream. The tested doses were selected based on previous findings reported by Radithia et al. [71].

##### Antimicrobial Assessment of Free LPEO and LPEO-CH-OA-NPs Cream After Vaginal Infection Induction

Swabs from each test rat (untreated and treated) were taken under sterile conditions and used to evaluate the number of living *Candida albicans* cells. Each swab was shaken with 1 mL of sterile saline solution (0.85% NaCl *w*/*v*), and 100 µL from each were cultured in nutrient agar plates, which were incubated at 30 °C for 24–48 h. After, the number of CFUs was assessed (CFU/mL).

##### Hematological and Biochemical Analysis

Vaginal secretions were collected on days 3 and 5 post-infection and cultured on Sabouraud dextrose agar.

At the end of the experiment, blood samples were collected through the jugular vein under light anesthesia for the assessment of the hematological profile, including hemoglobin (Hb) concentration (%), red blood cells (RBC) count, white blood cells (WBC) count, platelets count, hematocrit ratio, and reticulocytes count. All hematological analyses were performed using the hematology analyzer (Sysmex, Norderstedt, Germany). Blood samples were collected in test tubes and left for clotting. Then, the samples were centrifuged at 3000 rpm for 15 min to separate the serum. The serum was stored in sterile Cryotubes at −20 °C to be used for the biochemical analysis.

At the end of the experiment, all animals were euthanized, under anesthesia (intraperitoneal injection of ketamine 40 mg/kg), by cervical dislocation [72]. The vaginal tissues were then dissected, cleaned with PBS, and homogenized with a protease inhibitor and red blood cell lysate. The homogenate was centrifuged at 5000 g for 10 min, and the supernatant was collected and stored at −80 °C. Myeloperoxidase, interleukin 1β (IL-1β), malondialdehyde, and glutathione levels in the supernatant were measured using ELISA kits according to the manufacturer’s instructions. The OD value was measured with an enzyme-labelled instrument (Thermo Fisher, Waltham, MA, USA).

##### Histopathological Analysis

Specimens of vaginal tissue of rats from all experimental groups were harvested, fixed in neutral buffered formalin 10% (*v*/*v*), which were then dehydrated using ascending grades of ethyl alcohol, cleared in xylene, and embedded in paraffin. After, 4–5 µm thick sections were prepared and stained with hematoxylin and eosin staining solution for histopathological examination [73]. The slides were analyzed under a light microscope (BX43, Olympus, Tokyo, Japan) and photographed using the Cellsens dimension software 1.13 (Olympus) connected to the Olympus DP27 camera. The several vaginal tissues were examined thoroughly, and a lesion scoring was performed (+++: severe; ++: moderate; +: mild; -: none), as described by Wessam et al. [74].

### 3.8. Statistical Analysis

Experiments were conducted in duplicate or triplicate, and values were expressed as mean ± standard deviation (SD). GraphPad Prism (version 9.0.0.) was used to perform the statistical analysis, using Tukey’s test. Statistical significance was established at *p*-value < 0.05. Data processing and plot development were carried out using GraphPad Prism and Microsoft Excel 2016.

## 4. Conclusions

In conclusion, this work successfully developed and characterized chitosan–oleic acid nanoparticles loaded with lemon peel essential oil, as well as a topical cream formulation based on this nanosystem, as an innovative treatment for vulvovaginal candidiasis. The nanoparticles demonstrated high encapsulation efficiency, stability, and enhanced antimicrobial activity against *Candida albicans* and other microorganisms compared to free LPEO. Additionally, the LPEO-CH-OA-NPs incorporated into the topical cream exhibited sustained release and improved antifungal and anti-inflammatory effects in both in vitro and in vivo assessments, compared with the control groups. These results emphasize the potential of the developed formulation as an effective alternative for commercial VVC therapies, offering a promising solution to counteract drug resistance and improve treatment outcomes. Further research should focus on validating these findings and exploring their clinical applications.

## Figures and Tables

**Table 1:** Encapsulation efficiency, particle size, PDI, and zeta potential of the tested LPEO-CH-OA-NPs formulations (F1–F6).

Formulation	CH (g/L)	OA:LPEO	LPEO Content ± SD (%)	Particle Size ± SD (nm)	Zeta Potential ± SD (mV)	PDI ± SD
F1	5	2:1	71.02 ± 0.11	234.14 ± 0.11	65.4 ± 2.9	0.436 ± 0.020
F2	5	1:1	55.16 ± 0.18	345.40 ± 1.71	53.2 ± 0.8	0.498 ± 0.090
F3	5	1:2	68.52 ± 0.29	517.30 ± 2.23	59.5 ± 3.7	0.694 ± 0.110
F4	10	2:1	86.71 ± 0.97	442.54 ± 0.91	68.6 ± 4.4	0.248 ± 0.060
F5	10	1:1	84.62 ± 1.25	750.20 ± 3.48	99.5 ± 1.5	0.317 ± 0.010
F6	10	1:2	85.40 ± 2.74	757.70 ± 4.93	62.5 ± 1.9	0.773 ± 0.113

CH—chitosan. OA—oleic acid. LPEO—lemon peel essential oil.

**Table 2:** Minimum inhibitory concentration (MIC) and minimum microbicidal concentration (MBC) of LPEO and LPEO-CH-OA-NPs against the different testing microorganisms.

Sample (Initial Concentration)	*Staphylococcus aureus*		*Escherichia coli*		*Candida albicans*	
	MIC (µg/mL)	MMC (µg/mL)	MIC (µg/mL)	MMC (µg/mL)	MIC (µg/mL)	MMC (µg/mL)
LPEO (50 mg/mL)	312.2 ± 2.57	625.00 ± 5.23	156.25 ± 3.12	312.50 ± 4.81	39.06 ± 0.79	78.13 ± 1.09
LPEO-CH-OA-NPs (5 mg/mL)	39.06 ± 1.53	156.25 ± 6.21	78.13 ± 1.27	78.13 ± 2.04	78.13 ± 1.34	156.25 ± 1.84
LPEO-CH-OA-NPs (20 mg/mL)	19.53 ± 1.50	19.53 ± 0.99	19.53 ± 1.78	39.06 ± 0.96	9.78 ± 0.91	19.53 ± 1.01
LPEO-CH-OA-NPs (50 mg/mL)	9.77 ± 1.05	19.53 ± 1.42	9.78 ± 0.94	19.53 ± 1.65	9.78 ± 0.86	9.78 ± 0.92

**Table 3:** Tentative identification of LPEO-derived compounds by LC-ESI-UHR-QqTOF-MS.

ID	Proposed Name	Molecular Formula	Rt (min)	*m*/*z* Measured Mass [M + H]^+^	MS^2^ Fragments (*m*/*z*, % Relative Abundance)	Error (mDa)
1	Citronellic Acid	C_10_H_18_O_2_	6.4	153.1273	43 (100.0); 55 (54.1); 67 (49.8); 107 (18.3); 93 (16.8); 81 (15.4)	0.07
2	Eriodictyol	C_15_H_12_O_6_	7.9	289.0728	135 (100); 107 (78.7); 93 (33.8); 43 (7.1); 69 (20.3)	0.00
3	Linalool Oxide	C_10_H_18_O_2_	10.5	153.1274	43 (100); 55 (65.1); 81 (33.6); 67 (35.3); 91 (26.1)	0.04
4	1,8-Octanedioic Acid	C_8_H_14_O_4_	10.7	175.0816	133 (100); 89 (27.2); 177 (47.5); 45 (38.3)	0.17
5	3-Hydroxycapric Acid	C_10_H_20_O_3_	10.8	189.1483	107 (100.0); 43 (35.4); 135 (58.1); 81 (27.1); 153 (11.6)	0.23
6	Terpinolene	C_10_H_16_	17.1	137.1325	55 (100.0); 41 (61.0); 67 (74.2); 81 (71.0); 95 (40.1); 53 (26.8); 56 (30.8)	0.00
7	Mentha-1(7),8-dien	C_10_H_16_	17.5	137.1325	55 (100.0); 41 (63.6); 81 (64.5); 67 (78.1); 53 (24.8); 56 (39.1); 95 (46.6)	−0.10
8	Limonene	C_10_H_16_	19.0	137.1325	41 (36.2); 108 (100.0); 55 (33.6); 56 (68.1); 95 (10.3); 81 (20.2); 53 (15.0)	−0.20
9	α-Terpinene	C_10_H_16_	19.2	137.1325	108 (100.0) 41 (96.3); 55 (87.8); 81 (96.9); 67 (82.2); 53 (48.5); 95 (43.8)	−0.30
10	β-pinene	C_10_H_16_	19.5	137.1325	41 (100.0); 55 (90.1); 81 (91.3); 67 (52.4); 43 (52.2); 53 (35.6); 95 (38.1)	−0.20
11	Myrcene	C_10_H_16_	19.7	137.1325	41 (67.7); 55 (66.7); 81 (55.7); 43 (25.9); 67 (38.4); 95 (9.2); 53 (21.3); 108 (100.0)	−0.30
12	Camphene	C_10_H_16_	21.4	137.1325	121 (100.0); 55 (7.9); 43 (3.0); 136 (24.7); 53 (2.2); 120 (13.6); 41 (2.0)	0.10

**Table 4:** In silico docking results of 14-α-demethylase (PDB ID: 5TZ1), ∆-14-sterol reductase (PDB ID: 4QUV), 1,3-β-glucansynthase (PDB ID: 1EQC), and thymidylate synthase (PDB ID: 5UIV) by various ligands identified in LPEO to assess their antifungal activity. Interacting amino acids were assigned for metabolites showing high docking scores.

	Ligand (RMSD)							
	5TZ1		4QUV		1EQC		5UIV	
RMSD	0.2		0.7		0.5		0.8	
Compound	Binding Energy (kcal mol^−1^)	Interaction	Binding Energy (kcal mol^−1^)	Interaction	Binding Energy (kcal mol^−1^)	Interaction	Binding Energy (kcal mol^−1^)	Interaction
Miconazole Standard	−7.065	* HIS A: 377, * PHE A: 233, * LEU A: 376, * TYR A: 118, * ILE A: 231, * VAL A: 234, ** SER A: 507, ** TYR A: 64	−6.860	* TRP A: 256, * PHE A: 312, * LEU A: 346, * ARG A: 395, * LYS A: 259, * LYS A: 319, ** ASP A: 399, ** HIS A: 357, ** ARG A: 398	−6.979	* PHE A: 258, * TRP A: 373, * TYR A: 255, * TYR A: 317, ** GLU A: 292, ** ARG A: 309, ** ASP A: 145,	−6.121	* TYR A: 161, * TYR A: 100, ** ARG A: 39, ** LYS A: 17, ** GLU A: 159, ** ASP A: 13
Terpinolene	−4.760	ND	−4.880	ND	−4.645	ND	−5.099	* PHE A: 67, * LEU A: 51, * TYR A: 161, * PRO A: 37, * TYR A: 100
Mentha-1(7),8-dien	−4.620	ND	−4.780	ND	−4.459	ND	−5.148	* PHE A: 67, * LEU A: 51, * TYR A: 161, * PRO A: 37, * TYR A: 100
Limonene	−4.610	ND	−5.110	* HIS A: 357, * TRP A: 411, * TRP A: 352, * LYS A: 319, * LEU A: 347, * LEU A: 346, * ARG A: 323, * CYS A: 403	−4.672	ND	−5.520	* PHE A: 67, * LEU A: 51, * TYR A: 161, * PRO A: 37, * TYR A: 100
α-Terpinene	−4.560	ND	−5.240	* LYS A: 319, * LEU A: 346, * TRP A: 352, * HIS A: 357, * CYS A: 403	−4.538	ND	−5.609	* PHE A: 67, * LEU A: 51, * TYR A: 161, * PRO A: 37, * TYR A: 100
β-pinene	−4.700	ND	−4.180	ND	−4.917	* PHE A: 144, * PHE A: 258, * TYR A: 29, * LEU A: 304, * HIS A: 135, * TRP A: 363	−3.970	ND
Myrcene	−5.040	* LEU A: 121, * TYR A: 118, * LEU A: 376, * PHE A: 380, * HIS A: 377	−5.490	* HIS A: 357, * TRP A: 411, * TRP A: 352, * LYS A: 319, * LEU A: 347, * LEU A: 346, * PHE A: 322, * ARG A: 323	−4.808	* PHE A: 144, * PHE A: 258, * TYR A: 29, * LEU A: 304, * HIS A: 135, * TRP A: 363	−6.180	* PHE A: 67, * LEU A: 51, * TYR A: 161, * PRO A: 37, * TYR A: 100
Camphene	−4.530	ND	−4.150	ND	−4.814	* PHE A: 258, * TYR A: 255, * TRP A: 373, * TYR A: 29, * TRP A: 363, * PHE A: 144	−3.940	ND
Citronellic Acid	−5.510	* LEU A: 376, * CYS A: 470, * PRO A: 375, * MET A: 374, ** ARG A: 381, ** TYR A: 132	−5.144	* TRP A: 352, * LEU A: 346, * ARG A: 323, * TYR A: 414, * LYS A 319, ** LYS A: 319	−5.596	* PHE A: 144, * PHE A: 258, * TYR A: 29, * TYR A: 255, ** HIS A: 135, ** ASN A: 191, ** GLU A: 292	−6.160	* PRO A: 37, * TYR A: 161, * TYR A: 100, * PHE A: 67, ** ARG A: 92
Hydroxycapric Acid	−6.030	* LEU A: 376, * TYR A: 118, ** GLY A: 464, ** ARG A: 381, ** TYR A: 132, ** TYR A: 118	−5.355	** LYS A: 259, ** ARG A: 398, ** THR A: 255, ** TRP A: 256	−6.210	* PHE A: 144, * PHE A: 258, ** GLU A: 27, ** GLU A: 292, ** ASN A: 191, ** HIS A: 135	−6.320	* PRO A: 37, * PHE A: 67, * TYR A: 161, * TYR A: 100, ** ARG A: 92, ** GLN A: 167
Eriodictyol	−6.090	* GLY A: 307, * ILE A: 304, * CYS A: 470, * PRO A: 375, ** PRO A: 462, ** PRO A: 375	−6.130	* ASP A: 399, ** LYS A: 259, ** ARG A: 395, ** ARG A: 398	−7.077	* PHE A: 144, * TRP A: 363, * GLU A: 27, ** ARG A: 309, ** GLY A: 306, ** GLU A: 292, ** ASN A: 191	−5.947	* PHE A: 67, * PRO A: 37, * TYR A: 100, ** ARG A: 71, ** ARG A: 92, ** TYR A: 161, ** GLU A: 159
Linalool Oxide	−5.180	* TYR A: 118, * LEU A: 376, * HIS A: 377, ** MET A: 508	−4.520	ND	−5.704	* PHE A: 144, * PHE A: 258, * TYR A: 29, * HIS A: 135, * TRP A: 363, ** GLU A: 292, ** ASN A: 146	−5.250	* PHE A: 67, * LEU A: 51, * TYR A: 161, * PRO A: 37, * TYR A: 100
1,8-Octanedioic Acid	−5.450	* LEU A: 376, * CYS A: 470, ** TYR A: 118, ** TYR A: 132, ** ARG A: 381, ** PRO A: 375	−4.770	ND	−5.661	* PHE A: 144, ** GLU A: 27, ** HIS A: 135, ** ASN A: 191, ** LEU A: 304	−6.101	* PRO A: 37, * TYR A: 100, ** ARG A: 71, ** GLN A: 167

RMSD—root mean square deviation. * Hydrophobic alkyl and pi interaction. ** Hydrophilic conventional hydrogen bonding and Van der Waals interaction. ND—not determined.

**Table 5:** Histopathological lesion score of vaginal tissue alterations in all experimental rats.

Lesion Type	Negative Control	Positive Control	Topical Cream Control	Standard Drug (Miconazole)	LPEO 5%	LPEO 10%	LPEO Cream 5%	LPEO Cream 10%
Mucosa Necrosis	−	++	−	−	−	−	−	−
Hyperplastic Mucosa	−	+++	+	++	++	+	+	−
Inflammatory Cells Infiltration	−	+++	++	+	++	+	+	−
Submucosal Edema	−	++	++	+	+	+	+	+
Congested Blood Vessels	−	+++	−	−	−	−	−	−

“+”—mild lesion. “++”—moderate lesion. “+++”—severe lesion. “−“—no lesion observed.

**Table 6:** Composition of each formulation (F1–F6) tested for the development of the LPEO-loaded chitosan–oleic acid nanoparticles.

Formulation	CH (g/L)	OA:LPEO
F1	5	2:1
F2	5	1:1
F3	5	1:2
F4	10	2:1
F5	10	1:1
F6	10	1:2

CH—chitosan. OA—oleic acid. LPEO—lemon peel essential oil.

## Data Availability

The data generated and/or analyzed during the current study are available from the corresponding author on reasonable request.
